# Supplementary material for: Role of Healthcare Professionals and Sociodemographic Characteristics in COVID-19 Vaccination Acceptance among Uro-Oncology Patients: A Cross-Sectional Observational Study
Source: Vaccines (Basel). 2023 Apr 28;11(5):911. doi: 10.3390/vaccines11050911 (PMC10222021; doi:10.3390/vaccines11050911)
Supplement: Supplementary file 1 [file vaccines-11-00911-s001.zip › vaccines-2364237-supplementary.pdf]

QUESTIONNAIRE - Attitudes related to vaccination against COVID-19 in uro-oncology patients

**Sociodemographic data**	
Gender	1. Male<br>2. Female
Date of Birth	*day. month.year*
Permanent residence	1. In Capital city<br>2. Outside Capital city
The highest level of education	1. Elementary school or less<br>2. High School<br>3. Higher education
Are you a Healthcare Professional?	1. Yes<br>2. No
Number of household members	1. Living alone<br>2. Living with 1 member<br>3. Living with ≥2 members
Are any of the family members healthcare workers?	1. Yes<br>2. No
Do you have any other chronic diseases?	1. Yes<br>2. No
Of the listed, which chronic diseases do you have?<br>*(there can be more than one answer)*	1. Cardiovascular diseases<br>2. Diabetes mellitus<br>3. Obesity<br>4. Chronic obstructive pulmonary disease<br>5. Chronic renal failure
**COVID-19 vaccination**	
Have you been vaccinated against COVID-19?	1. Yes<br>2. No
Did you have enough vaccination information, given that you are being treated for a malignant disease	1. Yes<br>2. No
Have you consulted a doctor about vaccination?	1. Yes<br>2. No
Which doctors did you consult with<br>*(there can be more than one answer)*	1. Chosen Primary care physician<br>2. Urologist<br>3. Medical Oncologist<br>4. Other specialties
What did the doctors involved in your treatment advise you, given that you are being treated for a malignant disease	1. For vaccination<br>2. Against vaccination<br>3. Advise not received<br>4. Indeterminate
Have your household members been vaccinated?	1. Yes<br>2. No
What mostly influenced your decision to or NOT to vaccinate?<br>*(there can be more than one answer)*	1. Doctor's advice<br>2. Public media<br>3. Primary malignant disease<br>4. Family members<br>5. Personal beliefs<br>6. I don't want to answer
Have you previously contracted COVID-19 infection and when?	1. Yes, before systemic treatment started<br>2. Yes, during systemic treatment<br>3. No

QUESTIONNAIRE - Attitudes related to vaccination against COVID-19 in uro-oncology patients

for those who have been vaccinated	
How many doses of vaccine have you received?	1. Two doses<br>2. Three doses
Dates of all doses<br>1. 1st dose<br>2. 2nd dose<br>3. 3rd dose	 *day. month.year*<br>*day. month.year*<br>*day. month.year*
Have you hesitated to get the vaccine?	1. Yes<br>2. No
for those who have not been vaccinated	
Would you change your mind and get vaccinated now?	1. Yes<br>2. No
